# The risk of COVID-19 in Cushing's disease is independently related to disease activity (hypercortisolism) and obesity

**DOI:** 10.20945/2359-4292-2022-0313

**Published:** 2024-04-01

**Authors:** Bruna M. G. Mascarenhas-Nakano, Silvia R. Correa-Silva, Silvia M. R. Fracacio, Paola W. Brock, Rossella F. Dias, S. S. Binda Eduarda, Pedro F. Santos-Neto, Luiz H. C. Portari, Adriana Sanudo, Julio Abucham

**Affiliations:** 1 Universidade Federal de São Paulo Escola Paulista de Medicina Divisão de Endocrinologia São Paulo SP Brasil Unidade de Neuroendocrinologia, Divisão de Endocrinologia, Escola Paulista de Medicina, Universidade Federal de São Paulo (Unifesp), São Paulo, SP, Brasil; 2 Universidade Federal de São Paulo Escola Paulista de Medicina Departamento de Medicina Preventiva São Paulo SP Brasil Departamento de Medicina Preventiva, Escola Paulista de Medicina, Universidade Federal de São Paulo (Unifesp), São Paulo, SP, Brasil

**Keywords:** Cushing's disease, COVID-19, hypercortisolism, obesity

## Abstract

**Objective:**

To evaluate the cumulative incidence, risk factors, and outcomes of COVID-19 in patients with Cushing's disease (CD).

**Subjects and methods:**

In all, 60 patients with CD following up in our outpatient clinic answered via phone interview a questionnaire about the occurrence of COVID-19 infection documented by RT-PCR (including the diagnosis date and clinical outcome) and vaccination status. Clinical and biochemical data on disease activity (hypercortisolism) and comorbidities (obesity, diabetes mellitus, and hypertension) were obtained from the patients' electronic medical records. Risk ratios (RRs) of risk factors were obtained using univariate and multivariate analyses.

**Results:**

The cumulative incidence of COVID-19 in patients with CD during the observation period was 31.7%, which was higher than that in the general reference population (9.5%). The cumulative incidence of COVID-19 was significantly higher in patients with hypercortisolism (57% *versus* 17% in those without hypercortisolism, p = 0.012) and obesity (54% *versus* 9% in those without obesity, p < 0.001) but not in patients with hypertension or diabetes mellitus. On multivariate analysis, hypercortisolism and obesity were each independent risk factors for COVID-19 (RR 2.18, 95% CI 1.06-4.46, p = 0.033 and RR 5.19, 95% CI 1.61-16.74, p = 0.006, respectively).

**Conclusion:**

The incidence of COVID-19 in patients with CD was associated with hypercortisolism, as expected, and obesity, a novel and unexpected finding. Thus, correction of hypercortisolism and obesity should be implemented in patients with CD during the current and future COVID-19 outbreaks.

## INTRODUCTION

Hypercortisolism induces substantial changes in the entire immune system, affecting both cellular and humoral immunity (1). Cushing's syndrome is frequently associated with severe forms of common viral infections, including adenovirus, influenza, herpes simplex, herpes zoster, and cytomegalovirus (1). Cushing's disease (CD) has been associated with immunosuppression not only in the active phase of the disease but also after its remission (2).

Several studies have recently shown an increased risk of COVID-19 infection, hospitalization, and mortality in patients on glucocorticoid therapy, especially at high doses (more than 10 mg prednisone-equivalent doses) (3). In patients with CD, the only published study on the incidence of COVID-19 was conducted during the very first months of the pandemic and showed an increased cumulative incidence among patients with CD compared with a control group (4). However, the number of COVID-19 cases at that time was too small for statistical analysis after stratification of the patients according to disease control and other parameters.

Comorbidities like obesity, hypertension, and diabetes mellitus – which are highly prevalent in CD – have been shown to increase the severity of COVID-19 in the general population (5). More recently, a higher risk of COVID-19 infection has been shown in obese individuals (6).

The aim of this study was to evaluate the incidence, risk factors, and outcomes of COVID-19 in a cohort of patients with CD from February, 2020, to November, 2021 (before the arrival of the Omicron variant in Brazil).

## SUBJECTS AND METHODS

**Figure 1:**
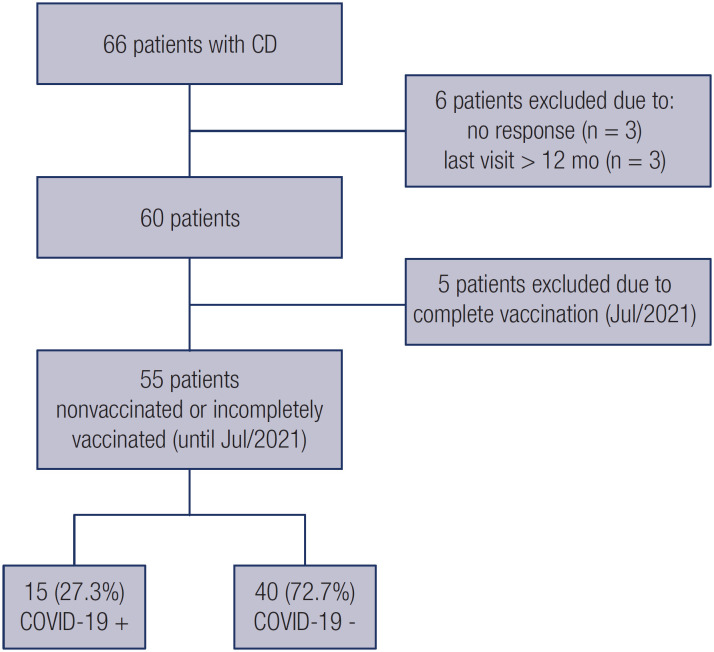
Flowchart outlining the enrollment of the study participants.

In all, 66 patients actively following up in our outpatient clinic with a diagnosis of CD confirmed by previous surgery were screened for enrollment (Figure 1). Six patients were excluded, including 3 who could not be contacted (nor could any family member) and 3 who had the last evaluation more than 1 year before.

**Figure 2:**
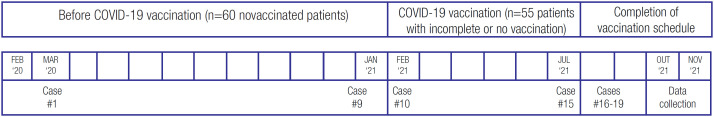
Schematic illustration of the COVID-19 pandemic and COVID-19 vaccination in Brazil during the inclusion period of the study.

A total of 60 patients (54 women, mean age 45.3 ± 10.7 years, age range 25-71 years) were enrolled in the study. From October 1 to November 30, 2021, all 60 patients (or their family members) answered a questionnaire via phone about the occurrence of COVID-19 infection documented by RT-PCR (including the date of diagnosis and clinical outcomes) and vaccination status (Figure 2). Clinical and biochemical data on disease activity and comorbidities in the last appointment (which had occurred at a median of 2.0 months [range 1-10 months, mean 2.9 ± 1.6 months] before the questionnaire) were obtained from the patients' electronic medical records. According to these evaluations (clinical features of hypercortisolism plus high nocturnal salivary cortisol and/or high urinary free cortisol [above the upper limit of normal] and/or nonsuppressible cortisol after 1 mg of dexamethasone), 15 (25%) patients had hypercortisolism and 45 (75%) had CD remission. Among the patients with active CD (*i.e.*, hypercortisolism), 13 (87%) were receiving medical treatment (9 with ketoconazole, 1 with cabergoline, and 3 with ketoconazole plus cabergoline) without control of hypercortisolism. Among those in remission, 9 (20%) were on glucocorticoid replacement (mean prednisone dose 4.5 ± 0.8 mg/day, range 2.5-5 mg/day), including 6 patients who had undergone bilateral adrenalectomy.

The 6 patients who were excluded after screening (out of the 66 screened patients) had a proportion of hypercortisolism (33%) and remission (67%) comparable to those in the enrolled patients, indicating no patient selection bias.

Data from the reference population were obtained from the website of the São Paulo State Health Secretary (https://www.seade.gov.br/coronavirus/). The RT-PCR results in the state of São Paulo (Brazil) from the beginning of the pandemic in March, 2020, to December, 2021, were notified by primary health care units and laboratories and were reported on the website at the link above.

The study was approved by the Human Research Ethics Committee of *Escola Paulista de Medicina* at *Universidade Federal de São Paulo* (CAAE: 56892522.7.0000.5505).

### Statistical analysis

The cumulative incidence of COVID-19 was calculated by dividing the number of patients with CD who reported COVID-19 infection by the total number of patients with CD during a defined time interval and was described as percentages with 95% confidence intervals (95% CIs). The resulting incidence was compared with the incidence of COVID-19 in the general population (obtained from https://www.seade.gov.br/coronavirus/) during the same period and in the same geographical area of the patients with CD. Normally distributed data are presented as mean ± standard deviation, and categorical data are presented as proportion (percent). Bivariate comparisons were performed using independent t-tests for continuous variables and Fisher's exact test for categorical ones.

To estimate the associations of COVID-19 with age, disease activity (hypercortisolism), and comorbidities (obesity, hypertension, and diabetes mellitus), we estimated risk ratios (RRs) and 95% CIs using a generalized linear model with Poisson distribution and a log link function with robust variance (7). The linear assumption between age or body mass index (BMI) and the respective dependent variable in the generalized linear model was assessed using the fractional polynomial method (8), which demonstrated that both variables could be included as linear covariates in the generalized linear model. In addition, multivariate analysis was performed to identify the variables independently associated with COVID-19.

The statistical analysis was performed using Stata/SE, version 15.1 (StataCorp LLC, College Station, TX, USA). A p value < 0.05 was considered statistically significant.

## RESULTS

The overall cumulative incidence of COVID-19 in the cohort during the entire observation period of 22 months, regardless of vaccination status, was 31.7% (19 out of 60, 95% CI 20.2-45%). This incidence was more than three times higher than that in the general reference population (9.5%) (https://www.seade.gov.br/coronavirus/) within the same area and during the same period. In Brazil, the first COVID-19 case was registered in February, 2020, and the vaccination only started in February, 2021. Thus, 55 patients were considered nonvaccinated (*i.e.*, received no vaccine or only one of two doses of the vaccine) up to July, 2021 (observation period of 18 months). Among the nonvaccinated patients, the cumulative incidence of COVID-19 during the study period was 27.3% (15 out of 55, 95% CI 16.1-41%), which was also more than three times higher than the incidence in the reference population (8.7%) (Table 1).

**Table 1:** Cumulative incidences, hospitalization data, and deaths due to COVID-19 in 55 nonvaccinated or incompletely vaccinated patients with Cushing's disease (CD) and in the reference population

	Patients with CD (n = 55)	Reference population (N = 46,649,132)
Cases	15 (27.3%)	4,057,868 (8.7%)
Hospitalization	7 (46.7%)	N/A
Hospitalization days	16(2-22)*	N/A
Death due to COVID-19	1 (6.7%)	134,646 (3.3%)
Death due to other causes	1 (6.7%)	N/A

N/A: not available.

* Median (range).

As shown in Table 2, disease activity (hypercortisolism) and obesity were each associated with COVID-19 infection. The incidence of COVID-19 infection was higher in patients with hypercortisolism (8 out of 14, 57.1%) compared with those in remission (7 out of 41, 17.1%; p = 0.012) and in patients with obesity (12 out of 22, 54.6%) compared with those without obesity (3 out of 33, 9.1%; p < 0.001). Age, hypertension, diabetes mellitus, types of drugs used to control diabetes mellitus or hypertension, hypopituitarism, or radiotherapy were not associated with COVID-19 (p > 0.05).

**Table 2:** Risk ratios (RRs) and corresponding 95% confidence intervals (95% CIs) for COVID-19 infection in 55 nonvaccinated or incompletely vaccinated patients with Cushing’s disease grouped according to disease activity and comorbidities

Covariates		Total	COVID-19 infection		RR	95% CI	p
			N	%			
Hypercortisolism							0.012
	No	41	7	17.1	1		
	Yes	14	8	57.1	3.35	1.48-7.55	
Obesity							<0.001
	No	33	3	9.1	1		
	Yes	22	12	54.6	6.00	1.91-18.84	
Hypertension							0.531
	No	20	4	20.0	1		
	Yes	35	11	31.4	1.57	0.58-4.29	
Diabetes mellitus							0.752
	No	36	9	25.0	1		
	Yes	19	6	31.6	1.26	0.53-3.02	

Patients with CD who had COVID-19 also had a higher mean BMI (36.0 ± 6.7 kg/m^2^) than those who did not have COVID-19 (28.2 ± 6.4 kg/m^2^; p < 0.001). In the generalized linear model, every 1.0 kg/m^2^ increment in BMI was accompanied by a 7% (95% CI 3.3-10.8%, p < 0.001) linear increase in the risk of COVID-19. No interaction or synergism between hypercortisolism and obesity was observed (p = 0.785).

**Figure 3:**
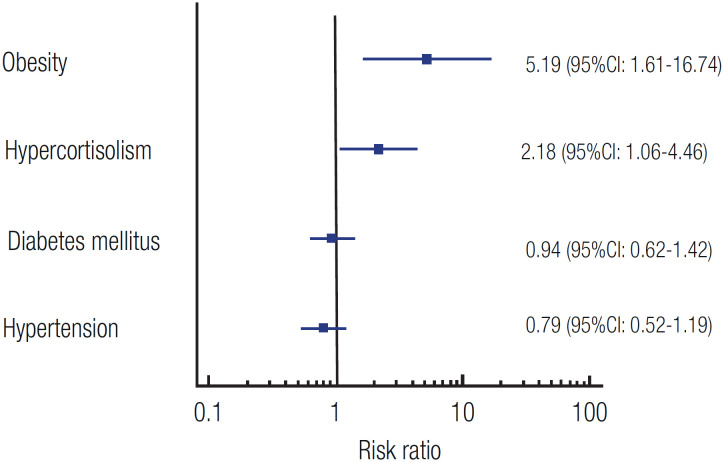
Risk ratios (RRs) for COVID-19 according to the presence of obesity, hypercortisolism, diabetes mellitus, and hypertension in patients with Cushing's disease.

On multivariate analysis, hypercortisolism and obesity emerged each as an independent risk factor for COVID-19 in patients with CD (RR 2.18, 95% CI 1.06-4.46, p = 0.033 and RR 5.19, 95% CI 1.61-16.74, p = 0.006, respectively), while hypertension and diabetes mellitus (both included as covariates) did not. These results are shown in Figure 3.

As shown in Table 1, nearly half (7 out of 15) of the patients with CD were admitted to the hospital when diagnosed with COVID-19. One patient (6.7%) died from COVID-19; this patient had not received any dose of the vaccine and had hypercortisolism, morbid obesity, hypertension, and diabetes mellitus.

Except for diabetes mellitus, which was significantly more prevalent in patients with CD who were hospitalized compared with those who were not hospitalized due to COVID-19 (5 out of 7 *versus* 1 out of 8, respectively, p = 0.04), no differences in age, hypertension, hypercortisolism, obesity or vaccination status (no vaccine *versus* one of two vaccine doses) were observed between patients who were or were not hospitalized due to COVID-19 (all p values > 0.28). 

## DISCUSSION

The results of our study show that the incidence of COVID-19 was three times higher in patients with CD who were either nonvaccinated or incompletely vaccinated compared with the general reference population within the same geographic area and during the same period. The incidence found is lower than – but comparable to – the five times higher incidence found in another cohort of patients with CD evaluated at the very beginning of the pandemic, before vaccination was available. However, the control group in that study was not composed of individuals from the general population but rather patients with nonfunctioning pituitary incidentalomas (4). Together, both data indicate a clearly increased susceptibility to COVID-19 among patients with CD. The apparent difference in incidences between the two studies may be due to different control populations but could also suggest a slight protection, even by incomplete vaccination, in most of our patients.

Our study is the first to stratify patients with CD according to disease activity and presence of comorbidities, which allowed us to analyze the relationship of these parameters with the risk of COVID-19 infection. Both disease activity and obesity emerged as independent risk factors for COVID-19 infection in patients with CD. Hypercortisolism has been classically associated with a compromised immune response and a high risk of infections, including viral ones. Many aspects of the immune response to viruses can be abnormal in patients exposed to glucocorticoid excess, including reduced natural killer cytotoxicity in virally infected cells, impaired complement activation pathways, low levels of B and T lymphocytes, and low phagocytic activity; these abnormalities may explain the high susceptibility of these patients to COVID-19 infection (9,10).

Although the associations between obesity and the severity of COVID-19 outcomes have been widely reported, only recently has obesity been shown to increase the risk of COVID-19 infection (6). The reason for this vulnerability of obese patients to COVID-19 infection is a matter of debate. Obesity is known to increase proinflammatory cytokines (including interleukin-6 and tumor necrosis factor alpha in the adipose tissue) and leptin (a proinflammatory adipokine) and to decrease adiponectin, leading to dysfunction of innate immunity (9). The chronic inflammatory state of obesity can also decrease the activation of macrophages in the presence of antigens. In our patients, we found no associations of diabetes mellitus or hypertension with COVID-19 infection.

Despite the small number of patients with diabetes mellitus, this condition was more prevalent in patients who were hospitalized due to COVID-19, which may reflect the well-known higher risk associated with this condition in COVID-19 outcomes.

One possible limitation of our study is that the high incidence of COVID-19 found in patients with CD in relation to the general population could have been overestimated due to an underestimation of COVID-19 infection rates in the general population. This could be due to the lack of available tests (RT-PCR) at the beginning of the pandemic in our country. However, data on COVID-19 in our patients with CD could also have been similarly influenced. At any rate, the only prevalence study of COVID-19 past infection in the general population (in the same geographic area and period, before vaccination, and using measurement of anti-SARS-CoV-2 antibodies) showed that 29.9% of the general population had positive anti-SARS-CoV-2 antibodies (11), a prevalence much higher than that provided by RT-PCR data. However, these data on past infections based on positive anti-SARS-CoV-2 antibodies in the general population cannot be directly compared with our data on symptomatic infection confirmed by RT-PCR in patients with CD.

In conclusion, COVID-19 in patients with CD was associated with hypercortisolism, as expected, but also with obesity, which is a novel and unexpected finding that has only recently been reported in the general population (6). Thus, correction of hypercortisolism, obesity, and preventive measures should be more aggressively implemented in patients with CD during the current and future COVID-19 outbreaks.
